# Cu(I)-thioether coordination complexes based on a chiral cyclic β-amino acid ligand

**DOI:** 10.1038/s42004-023-01055-5

**Published:** 2023-11-16

**Authors:** Jihee Lee, Jaewook Kim, Hongil Jo, Danim Lim, Jungwoo Hong, Jintaek Gong, Kang Min Ok, Hee-Seung Lee

**Affiliations:** 1 Department of Chemistry, Korea Advanced Institute of Science and Technology (KAIST), 291 Daehak-ro, Yuseong-gu, Daejeon 34141 Republic of Korea; 2 Center for Multiscale Chiral Architectures (CMCA), KAIST, 291 Daehak-ro, Yuseong-gu, Daejeon 34141 Republic of Korea; 3 Department of Chemistry, Sogang University, 35 Baekbeom-ro, Mapo-gu, Seoul 04107 Republic of Korea; 4 Present Address: Department of Chemistry Education, Sunchon National University, 255 Jungang-ro, Suncheon-si, Jeollanam-do 57922 Republic of Korea

**Keywords:** Solid-state chemistry, Ligands, Optical materials, Coordination chemistry

## Abstract

Coordination complexes, particularly metalloproteins, highlight the significance of metal-sulfur bonds in biological processes. Their unique attributes inspire efforts to synthetically reproduce these intricate metal-sulfur motifs. Here, we investigate the synthesis and characterization of copper(I)-thioether coordination complexes derived from copper(I) halides and the chiral cyclic β-amino acid *trans*-4-aminotetrahydrothiophene-3-carboxylic acid (ATTC), which present distinctive structural properties and ligand-to-metal ratios. By incorporating ATTC as the ligand, we generated complexes that feature a unique chiral conformation and the capacity for hydrogen bonding, facilitating the formation of distinct geometric structures. Through spectroscopic analyses and density functional theory (DFT) calculations, we studied the complexes’ optical properties, including their emission bands and variable second-harmonic generation (SHG) efficiencies, which vary based on the halide used. Our findings underscore the potential of the ATTC ligand in creating unusual coordination complexes and pave the way for further investigations into their potential applications, particularly within materials science.

## Abstract (web summary)

Cu(I)-thioether complexes have been widely studied as metalloprotein mimics, but limited structural geometries have been reported to date. Here, the authors demonstrate that a chiral cyclic β-amino acid ligand affords Cu(I)-thioether complexes with chiral conformations and the capacity for hydrogen bonding, offering unusual geometric structures and metal-to-ligand ratios.

## Introduction

Coordination complexes represent a fundamental category of compounds ubiquitously found in nature, executing crucial functions in metabolism, transport, and redox catalysis within living organisms^1–4^. Notably, within the vast array of natural coordination complexes, metalloproteins distinguish themselves as a particularly significant group, including three subclasses: iron-sulfur proteins, cytochromes, and blue copper proteins. The distinctive characteristic of these proteins is the presence of metal-sulfur bonds, which are instrumental in the operation of biological metal centers. The role of the sulfur-donor ligand in these complexes extends to modulating the activity of the metal center and facilitating substrate binding, acid-base activity, or redox processes, all of which are indispensable for the efficient functioning of the active site^5^.

Recognizing the significant role of metalloproteins in biology, the scientific community has turned its focus toward the synthesis and characterization of artificial complexes that replicate these metal-sulfur motifs^6,7^. A considerable number of these studies have given special attention to copper ions and artificial sulfur-donor ligands derived from the thioether moiety of methionine. The selection of copper as the metal of choice stems from its cost-effectiveness as well as its enticing properties that hold promise for prospective bioapplications^8–11^. Moreover, with its thioether functional group, methionine is a preferable candidate for experimentation due to its independence from pH variations, unlike certain amino acids such as histidine and cysteine^12^. Considering these benefits, investigating Cu(I)-thioether complexes as biomimetic models emerges as a promising field within biomimetic chemistry^13–15^.

Cu(I)-thioether complexes exhibit several defining characteristics. Primarily, the coordination number for these Cu(I) complexes typically ranges from 2 to 4, resulting in linear, trigonal planar, or tetrahedral geometries. Moreover, the prevalent ratio of Cu(I) precursors to thioether ligands is 2:1 or 1:1, engendering a broad spectrum of geometric variations^16–21^. Interestingly, the valence electron pairs from a thioether ligand can serve dual roles: as a bridging ligand and a terminal ligand, providing potential avenues for crafting unique structures^22,23^. Nonetheless, despite this potential for structural variety, the range of unique structures in existing studies is somewhat limited. The inherent chelating effect of coordination compounds often stabilizes the structure, thus challenging the synthesis of monodentate thioether complexes^24,25^. Furthermore, the configuration at the sulfur atom can readily reverse due to the low barrier, which complicates the synthesis of chiral monodentate thioether complexes^26^. Few studies have attempted to confront these impediments to structural diversity. For instance, a study by Henline and colleagues demonstrated how the Cu(I) iodide-tetrahydrothiophene system could adopt diverse structures under varying experimental conditions, such as reaction temperature and stoichiometry^27^. Similarly, Solari and coworkers uncovered the impact of solvent on the Cu(I) coordination structures^28^. Despite these efforts, these approaches have not yet enabled an atypical ratio of Cu(I) precursors to thioether ligands or monodentate bonding.

We have recently disclosed the synthesis of a chiral β-amino acid based on tetrahydrothiophene, named *trans*-(3*S*,4*R*)-4-aminotetrahydrothiophene-3-carboxylic acid (*trans*-ATTC)^29,30^. This compound lends itself to diverse post-synthetic modifications. Recognizing the potential of ATTC to act as an effective metal-coordinating ligand, we hypothesized it could be employed to synthesize a metal-ATTC complex. In this study, we present a method for creating monodentate Cu(I)-thioether complexes that feature unique ligand-to-metal ratios and distinctive structures. Our approach incorporates a synthetic β-amino acid derivative as the ligand, which was intentionally designed to possess three essential attributes: (1) a thioether functional group to facilitate coordination with metal ions; (2) a cyclic structure to establish a chiral conformation; and (3) the ability to form hydrogen bonds, which can lead to the formation of unconventional geometric structures. By merely altering the halide in CuX (X = Cl, Br, and I), we could synthesize either monodentate complexes or a coordination polymer under ambient conditions. Interestingly, the chirality of the ligand’s S-donor moiety may influence the handedness of the resulting monodentate complexes. The hydrogen-bonding capability of our synthetic β-amino acid derivative can further induce an atypical stacking of the coordination polymer. As a result, the Cu(I) halide complexes formed with our specially designed ligand are expected to present not only unique structural attributes but also singular optical properties, such as circular dichroism (CD) and second-harmonic generation (SHG). This research contributes to the understanding of complex formation and paves the way for future investigations into the utility of these structures.

## Results and discussion

### Synthesis and Crystal Structures of Cu(I) complexes

**Fig. 1:**
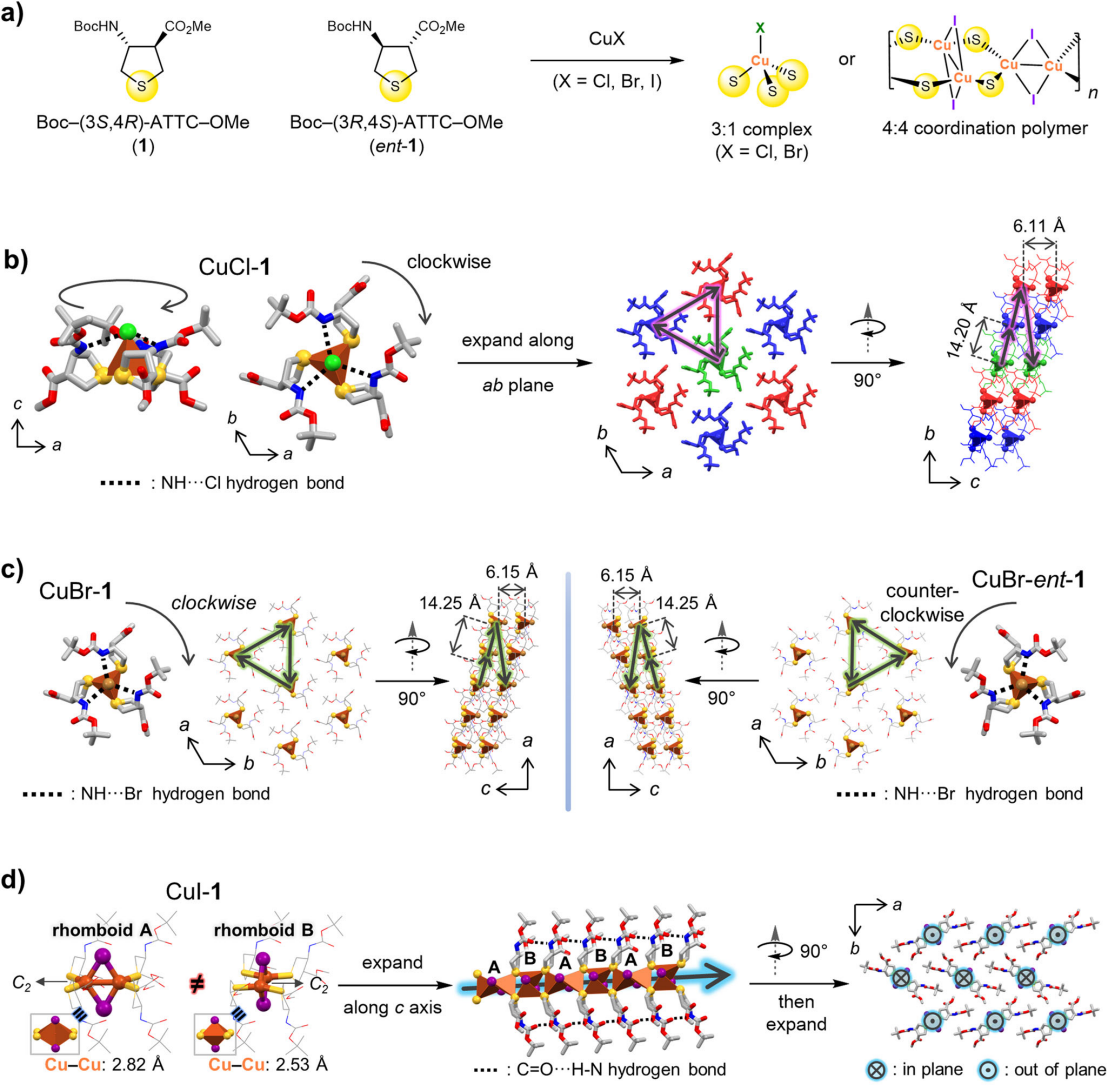
Synthesis and crystal structures of ATTC-Cu complexes. **a** Chemical structures of *trans*-4-aminotetrahydrothiopehen-3-carboxylic acid (ATTC) monomer **1** and corresponding Cu(I) complexes explored in this study. **b** The crystal structure of CuCl-**1** represents a 3:1 ligand-metal discrete complex with *C*_*3*_ tetragonal geometry, which is stabilized by hydrogen bonding (indicated by black dotted lines) between the carbamate N–H and chloride anion. The three molecules of **1** exhibit a clockwise arrangement. **c** CuBr-**1** and CuBr-*ent*-**1** crystal structures resemble CuCl-**1** but display slight elongation. **d** Crystal structure of CuI-**1** incorporates two distinct types of Cu_2_I_2_ rhomboid complexes with *C*_2_ symmetry. Linear 4:4 coordination polymer (highlighted by a gray arrow against a blue backdrop) along the *c*-axis is stabilized through hydrogen bonding (marked by black dotted lines). The two types of rhomboids alternate in their arrangement. The three-dimensional packing structure of CuI-**1** features a layered structure where each layer of coordination polymers is oriented in the opposite direction.

The combination of Boc–*trans*-ATTC–OMe (**1** and *ent*-**1**) with Cu(I) precursors in acetonitrile quickly precipitated white (For the synthetic procedure, see the Methods and Supplementary Methods sections. For NMR spectra, see Supplementary Figs. 1–6), needle-like solids suitable for X-ray diffraction analysis (Fig. 1a, for crystallographic information Supplementary Table 1). The crystal structure of CuCl-**1** revealed a 1:3 metal-ligand complex with a *C*_3_ tetrahedral geometry and *R*3 space group. This arrangement deviates from the previously established 1:1 or 1:2 complexes between Cu(I) and sulfide ligands (Fig. 1b, Supplementary Data 1)^16–21^. Within the single discrete complex, three **1** ligands coordinate with the Cu(I) cation in a η^1^-mode as terminal ligands. Intriguingly, three **1** molecules, when viewed from the *b*-axis, align in a clockwise direction. This alignment is stabilized by three hydrogen bonds between the carbamate N–H and chloride anions. (See Supplementary Table 2 for detailed bond length and bond angle data). The complexes demonstrate a layer-by-layer structure, with Cu-Cu distances measuring 6.106 Å along the *c*-axis and 14.199 Å along the [12-1] direction. The **1** complex with CuBr exhibited a structure similar to the CuCl structure, except for slightly elongated bond lengths and distances between complexes (Fig. 1c, Supplementary Table 1, Supplementary Data 2, 3). Intramolecular hydrogen bonds between carbamate N–H and the bromide anion were also noted. The orientation of the three **1** molecules depends on the stereochemistry of the ATTC ligand, with clockwise and counterclockwise directions for CuBr-**1** and CuBr-*ent*-**1**, respectively.

On the other hand, the CuI-**1** complex was found to have an orthorhombic metal-organic coordination polymer (MOCP) structure with a *P*2_1_2_1_2 space group (Fig. 1d, Supplementary Table 1, Supplementary Data 4). The metal-ligand ratio here was 4:4, distinct from the complexes with chloride and bromide anions. Two types of Cu_2_I_2_ rhomboids alternated in alignment, linked by two μ^2^-mode bridging ATTC ligands (See Supplementary Table 1 for detailed bond length and bond angle data). The CuI-**1** coordination polymer also exhibited cuprophilic interactions, with perpendicular Cu-Cu pairs bridged by iodide in a μ^2^-mode, showing bond lengths of 2.823 and 2.531 Å depending on the rhomboid type. Each coordination polymer chain extended indefinitely along the *c*-axis, reinforced by hydrogen bonds between the **1** ligands. It is hypothesized that the dual role of ATTC as a hydrogen bond donor and acceptor contributes to the formation of a Cu(I) complex with a unique structure, such as a 1:3 metal-ligand discrete complex or 4:4 coordination polymer^27^. Additionally, scanning electron microscope (SEM) and powder X-ray diffraction (PXRD) analyses confirmed that all Cu(I)-ATTC complexes possessed uniform, crystalline structures irrespective of the stereochemistry of **1** (Supplementary Figs. 7, 8). Discrepancies between calculated and experimental PXRD are attributed to the morphology of crystals (for a detailed discussion on this topic, refer to Supplementary Figs. 9, 10, and the ensuing Supplementary Discussion)^31,32^.

### Fourier-Transform Infrared (FT-IR) spectroscopy

**Fig. 2:**
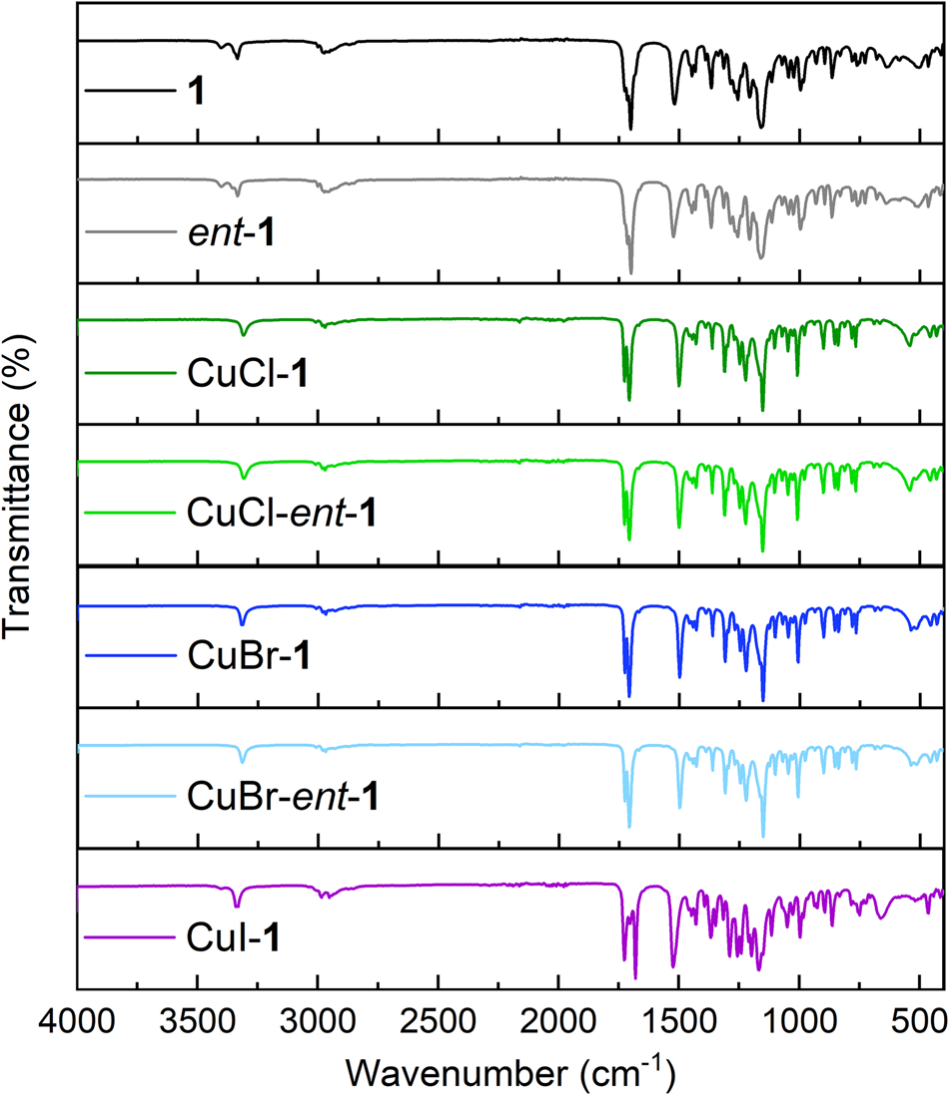
FT-IR spectra. FT-IR spectra of **1,** *ent*-**1**, and related CuX complexes (X = Cl, Br, and I).

The FT-IR spectra of all synthesized compounds, including the ATTC ligand and its Cu(I) coordination complexes, displayed characteristic bands at ~3320, 2950, and 1510 cm^−1^, attributable to the N–H stretching mode, C–H stretching mode, and the N–H bending mode of the ATTC ligand, respectively (Fig. 2). The spectrum region of 1650–1750 cm^−1^ revealed two absorption bands likely indicative of the C=O stretching mode in the ester and carbamate functionalities of the ATTC ligand. The bands within the range of 1000–1300 cm^−1^ could be associated with the C–O stretching mode of the ester and carbamate group of the ATTC ligand. The Cu–S absorption bands, typically observed around 600 cm^−1^ with minor intensity, overlapped with other peaks in the fingerprint region, complicating accurate assignment. Nevertheless, the spectral range of 500–525 cm^-1^ in the FT-IR spectra of CuX-ATTC (X = Cl, Br) could be ascribed to the Cu–X (X = Cl, Br) bond, considering the higher band polarity of Cu–Br compared to the Cu–Cl bond. For the CuI-**1** spectrum, the range of 625–675 cm^−1^ might be indicative of the Cu–Cu interaction or Cu–I bond.

### Thermogravimetric (TG) analysis

**Fig. 3:**
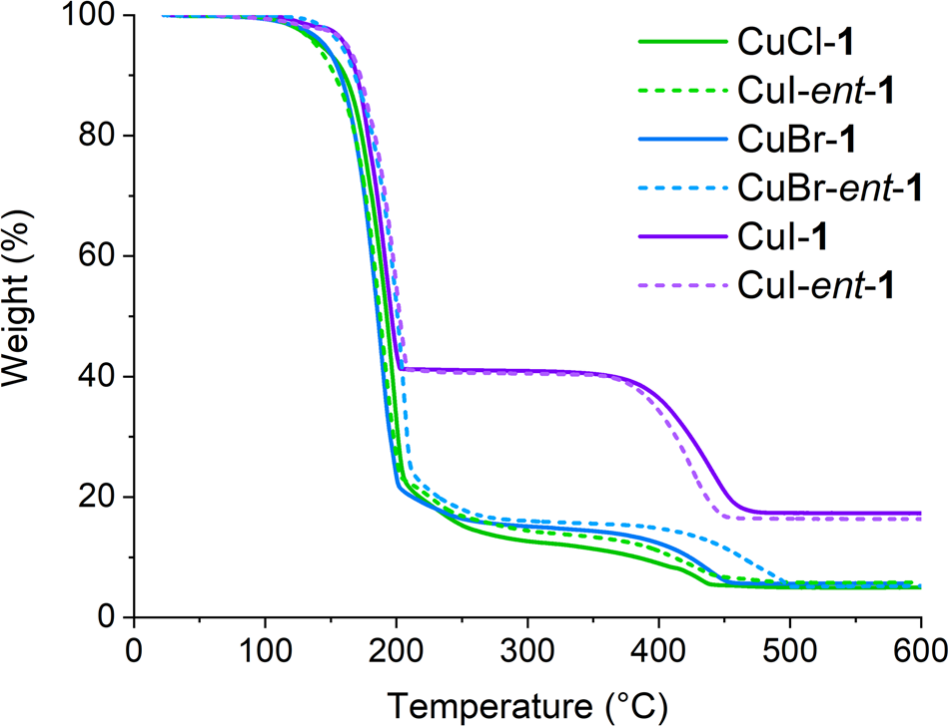
TGA analysis. Thermogravimetric (TG) analysis of CuX-**1** complexes (X = Cl, Br, and I).

The thermal properties of CuX-ATTC complexes (X = Cl, Br, I) were investigated using TG analysis, with temperatures spanning from room temperature to 600 °C under flowing air (Fig. 3). All complexes initiated a significant mass loss at around 180 °C, suggesting the thermal decomposition of ATTC ligands. A secondary mass loss at around 400 °C, likely attributable to Cu(II) oxide formation through further air oxidation, was also noted (Supplementary Fig. 11). Due to differences in metal-ligand ratio, the residual sample mass of the CuI-ATTC complex was approximately three times that of the chloride and bromide complexes.

### UV-Vis Diffuse Reflectance (UV-Vis DR) spectroscopy

**Fig. 4:**
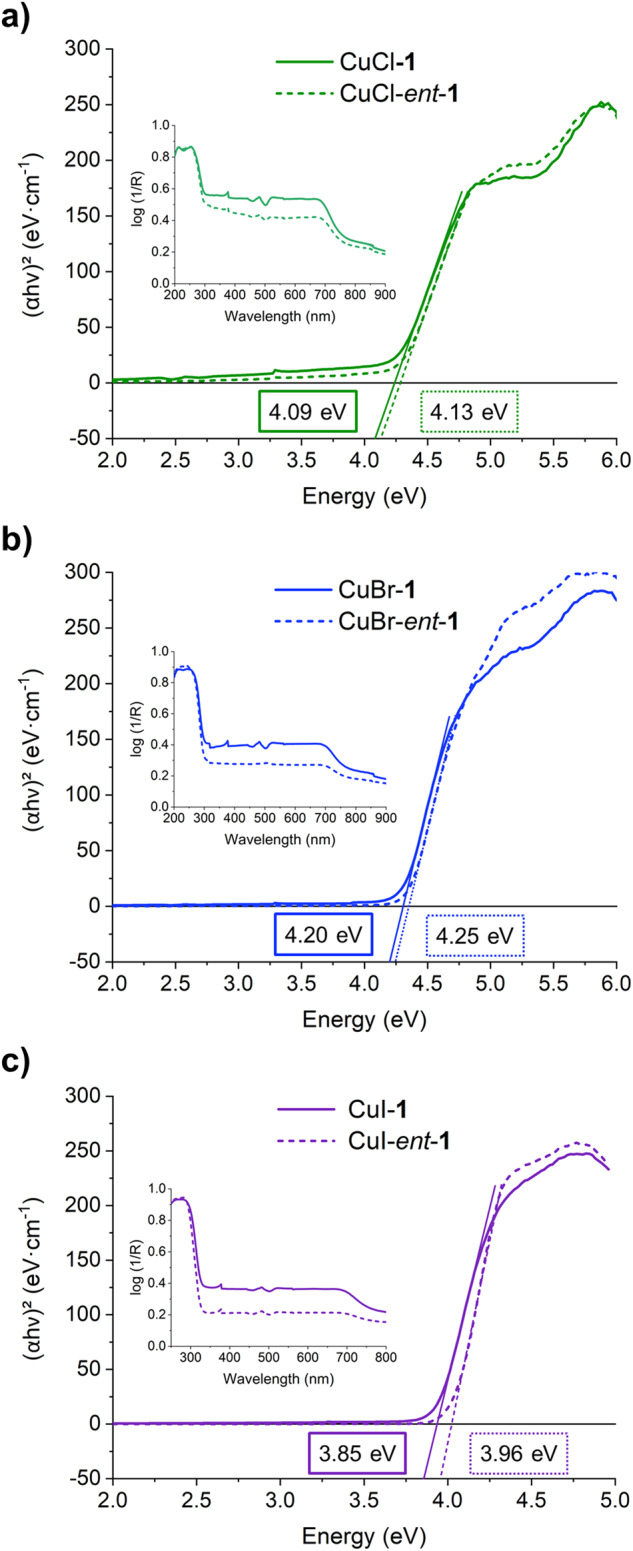
Estimation of the band gap energy. Kubelka-Munk plots for the estimation of the band gap energy of **a** CuCl-**1** and CuCl-*ent*-**1,** **b** CuBr-**1** and CuBr-*ent*-**1**, and **c** CuI-**1** and CuI-*ent*-**1**. (inset) UV-Vis diffuse reflectance spectra of **1** and CuX-**1** complexes.

The optical band gaps of the complexes were also examined utilizing UV-Vis DR spectra and the Kubelka-Munk equation (Fig. 4). The determined optical band gap energy values for CuCl-**1**, CuCl-*ent*-**1**, CuBr-**1**, CuBr-*ent*-**1**, CuI-**1**, and CuI-*ent*-**1** were 4.09, 4.13, 4.20, 4.25, 3.85, and 3.96 eV, respectively. The optical band gap energy for all coordination compounds exhibited a minor difference, but these differences were minimal and might be influenced by changes in the halide and the absolute configuration of the ligand.

### Density Functional Theory (DFT) calculations

**Fig. 5:**
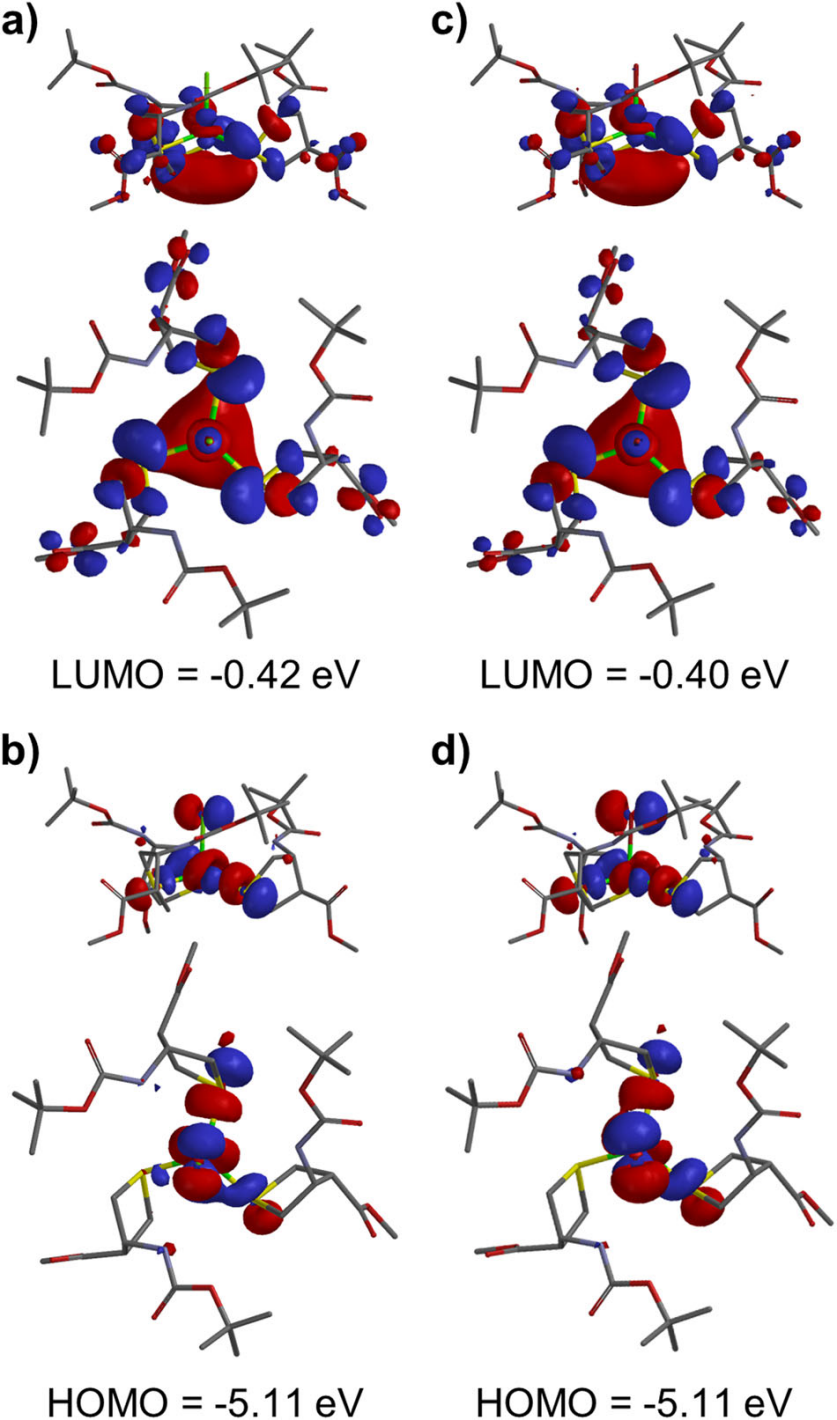
Visual representation of DFT-calculated frontier molecular orbitals for CuX-1 complexes. The figures depict (**a**) side and top views of LUMO for CuCl-**1**. **b** side and top views of HOMO for CuCl-**1**. **c** side and top views of LUMO for CuBr-**1**. **d** side and top views of HOMO for CuBr-**1**.

Using the crystal structures of CuCl-**1** and CuBr-**1**, highest occupied molecular orbital (HOMO) and lowest unoccupied molecular orbital (LUMO) energy values were calculated. For CuCl-**1**, the HOMO and LUMO energy values were −5.11 and −0.42 eV, respectively, creating an energy gap of 4.69 eV (Fig. 5). Similarly, for CuBr-**1**, the HOMO and LUMO energy values were −5.11 and −0.40 eV, respectively, yielding an energy gap of 4.71 eV. These calculated gaps were found to be larger than the measured optical band gaps, though the trend of a larger gap for CuBr–ATTC than for CuCl–ATTC remained consistent. The HOMO of CuX–ATTC (X = Cl, Br) complexes is predominantly composed of the 3p orbital of sulfur in the ATTC ligand, the 3d_*xy*_ and 3d_*yz*_ orbitals of the Cu center, and the 3p (for Cl) or 4p (for Br) orbital of the halide ligand. The LUMO is mainly made up of the σ* molecular orbitals of the ATTC ligands, arising from the combination of C_2*p*_ and S_3*p*_ orbitals and overlapping with each other.

### Solid-State CD Spectroscopy

**Fig. 6:**
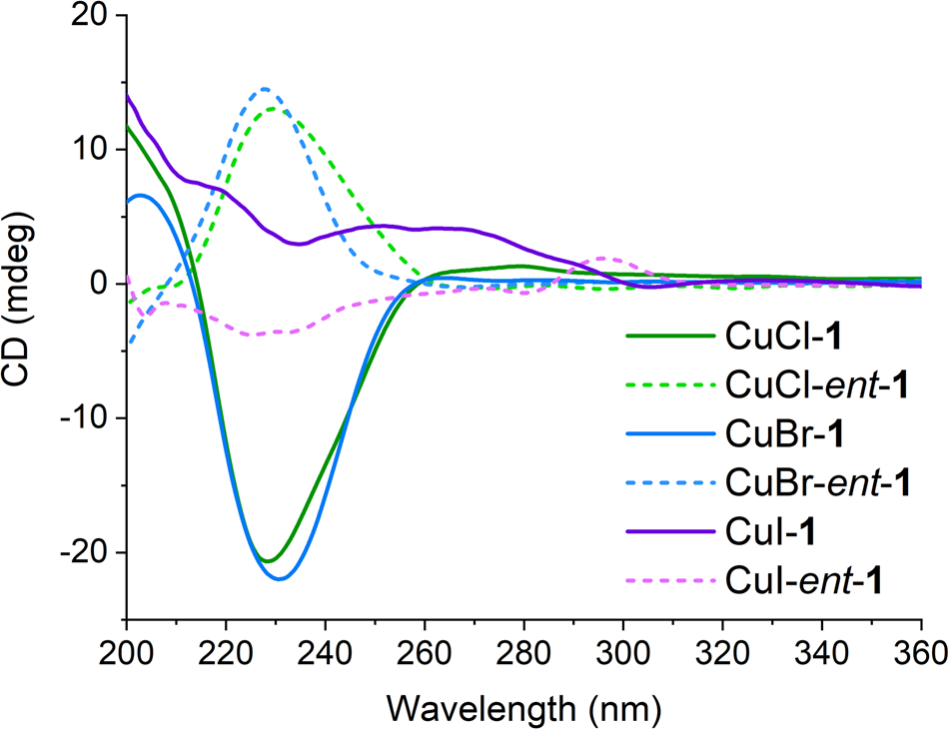
Solid-state CD spectra. Solid-state circular dichroism (CD) spectra of the CuX-ATTC complexes (X = Cl, Br, I).

Solid-state CD analysis was performed to ascertain the chiroptical property of CuX-ATTC complexes (Fig. 6). Notably, CuCl-ATTC and CuBr-ATTC complexes demonstrated strong CD signals in the 210–260 nm range, with a negative or positive Cotton effect around 230 nm for **1** or *ent*-**1**, respectively. In contrast, CuI-ATTC complexes showed no significant Cotton effect in the region, similar to CuCl and CuBr complexes.

### Photoluminescence (PL) Spectroscopy

**Fig. 7:**
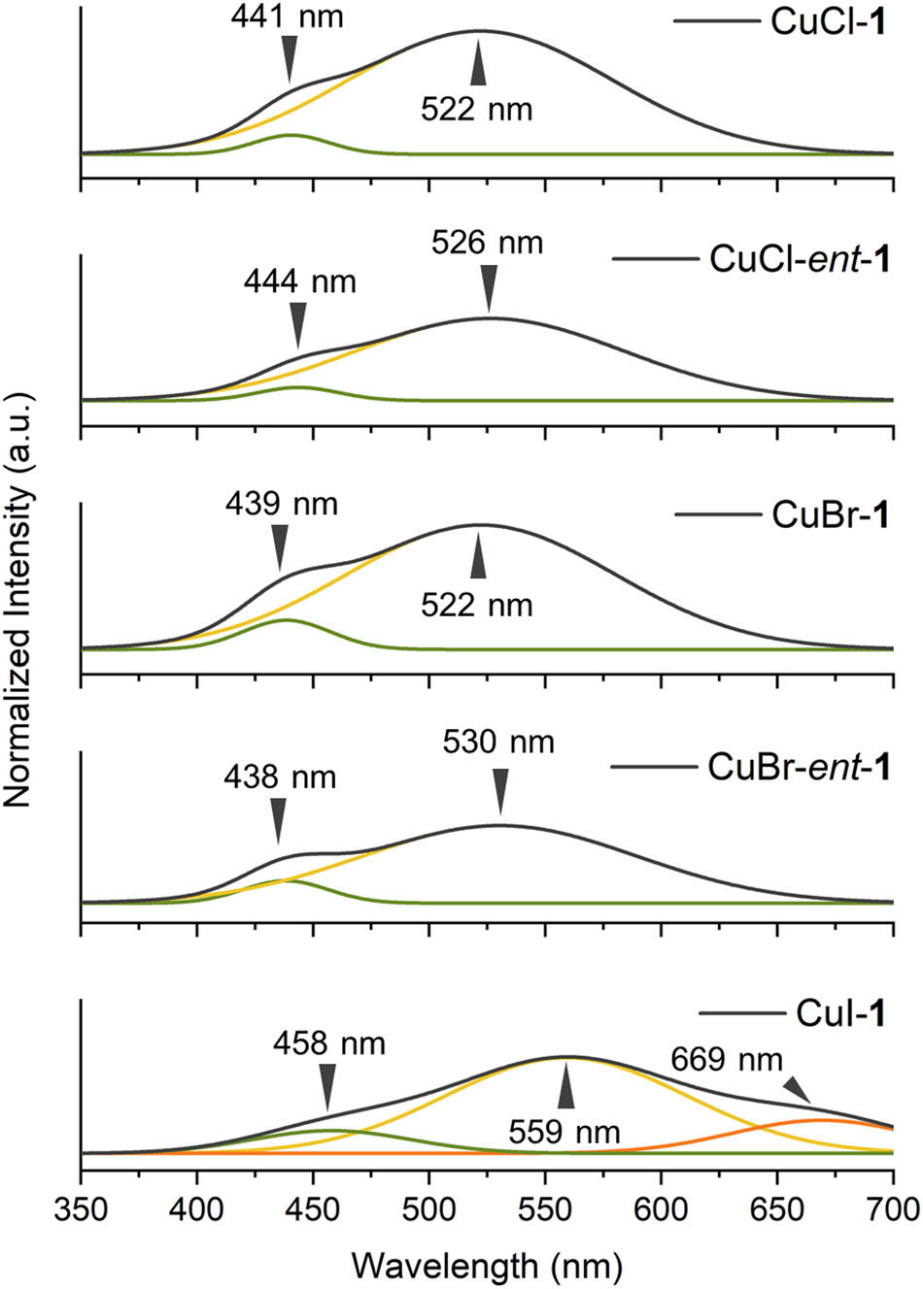
Solid-phase PL spectra. Smoothed and deconvoluted solid-phase photoluminescence (PL) spectra for the CuX-**1** and CuX-*ent*-**1** complexes (X = Cl, Br, I) at 293 K. The overall spectra are illustrated in black, and the deconvoluted spectra are in green, yellow, and orange.

The photoluminescence properties of the CuX-**1** (X = Cl, Br, I) complexes at room temperature are summarized in Fig. 7 (Supplementary Fig. 12, Supplementary Table 3). All complexes with the ATTC ligand exhibited an emission band around 430 nm, likely due to the proximity of the carbonyl group between the ATTC ligands^33^. An additional emission band in the 520–560 nm range was noted across all samples, potentially due to cluster-centered (CC) emission and halide-to-metal charge transfer (XMCT) behavior. Particularly, the emission spectrum of the CuI–ATTC complex demonstrated another emission band at ~670 nm, likely related to the influence of Cu–Cu interactions on the luminescence properties of Cu_2_I_2_ rhomboids, the locally excited (LE) emission band^34^.

### Second-Harmonic Generation (SHG) Spectroscopy

**Fig. 8:**
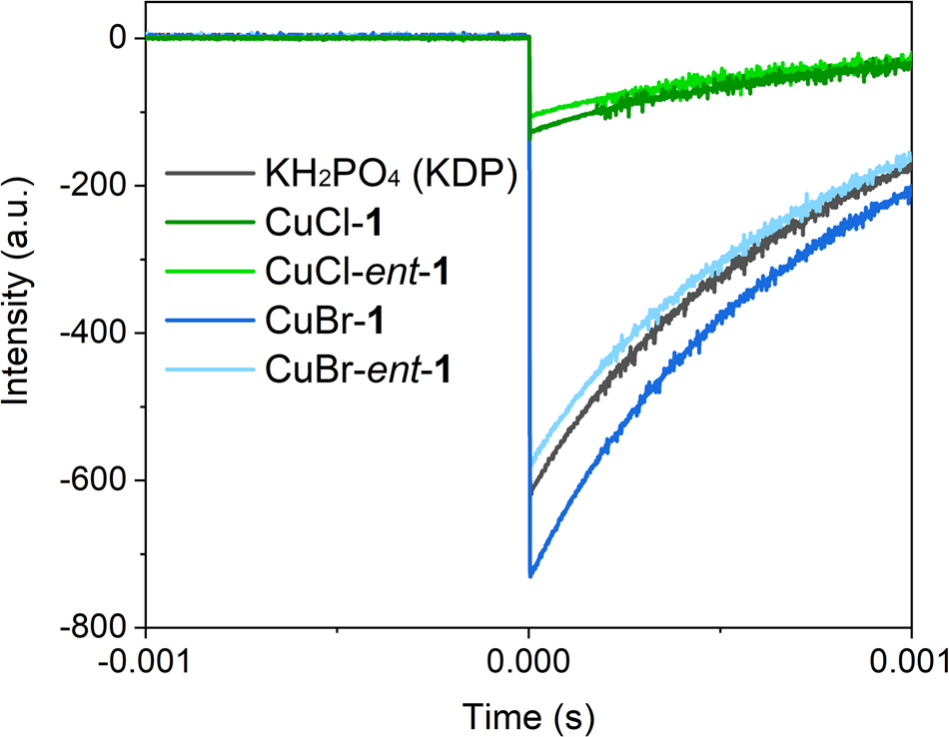
SHG Spectroscopy. Oscilloscope traces showing the intensity of the second-harmonic generation (SHG) signal for the CuX-**1** and CuX-*ent*-**1** complexes (X = Cl, Br).

The SHG efficiencies of complexes exhibiting the noncentrosymmetric (NCS) space group, *R*3, were examined^35^. Despite similar molar densities among CuBr-**1**, CuBr-*ent*-**1**, and CuCl-**1** (1.569, 1.568, and 1.585 mmol ·cm^−^^3^, respectively), the SHG intensity for ungraded samples of CuBr-**1** and CuBr-*ent*-**1** was found to be on par with the reference material, potassium dihydrogen phosphate (KDP). In contrast, CuCl-**1** and CuCl-*ent*-**1** registered about 0.3 times the intensity of KDP (Fig. 8). To understand these observations, the dipole moments of CuS_3_X (X = Cl and Br) units were calculated using the DFT method. Since CuCl-ATTC and CuBr-ATTC maintain 3-fold symmetry along the Cu–X bond, the cumulative dipole moment from the three ATTC units aligns with this bond (Supplementary Fig. 13). Compared to Cu–Cl, the larger polarizability of the Cu–Br bond is due to the bromide anion’s greater ionic radius, leading to a higher SHG efficiency for CuBr-ATTC despite similar dipole moments for both complexes. The alignment of CuX-ATTC’s dipole moment along the *c*-axis further enhances its SHG efficiency (Supplementary Fig. 14)^36^. Consequently, the symmetry of CuX-ATTC’s molecular and packing structures positively influences the SHG efficiency. Finally, the calculated local dipole moments of CuS_3_Cl and CuS_3_Br were 0.28–0.35 D and 3.14–3.15 D, respectively, with the larger dipole moment for CuS_3_Br attributed to the longer charge separation resulting from the more polarizable, larger ionic radius of Br^−^.

## Conclusion

In summary, this study has contributed to understanding coordination complexes by developing and analyzing Cu(I)-ATTC complexes. These complexes show unique features such as chiral conformation and hydrogen bonding, hinting at creating unusual geometric structures. The research further reveals the impact of halide changes on the properties of the complexes. Through detailed spectroscopic analyses and DFT calculations, we have identified key functional groups and observed distinct emission bands, along with differences in second harmonic generation efficiencies. These findings highlight the usefulness of the ATTC ligand in creating coordination complexes with interesting structures and optical properties. This research deepens our knowledge of coordination chemistry and lays a foundation for exploring the potential applications of these compounds, especially within the field of nonlinear optical materials^37–40^ and broader areas of materials science.

## Methods

### Materials

All reactions were performed in oven-dried round-bottom flasks under N_2_ atmosphere. Unless otherwise noted, all commercially available reagents and solvents were used without additional purification. Starting materials and reagents were purchased from Sigma-Aldrich and Tokyo Chemical Industry (TCI). Acetonitrile was distilled under CaH_2_ and stored under 4 Å molecular sieve, and degassed by bubbling nitrogen gas for 30 min before use. Dimethylformamide (DMF) was vacuum distilled and stored under 4 Å molecular sieve. High-purity water was generated by the Millipore Milli-Q apparatus. Analytical thin-layer chromatography (TLC) was performed using Merck (Darmstadt, Germany) silica gel 60 F_254_ glass plates. TLC plates were visualized using a 254 nm UV lamp, acidic ninhydrin, or an anisaldehyde solution. Purification of the crude reaction mixture by column chromatography was performed using Merck silica gel 60 (230–400 mesh).

### General procedure for the synthesis of CuX-1 complexes

150 mM of CuX (X = Cl, Br, and I) and 400 mM of **1** in acetonitrile stock solutions were prepared. On the 4 mL vial, 1.0 mL of CuX stock solution and 1.0 mL of **1** stock solution were mixed. After closing the cap tightly, the mixture was stirred at room temperature. White precipitate formed immediately upon stirring. Solvents were decanted, and the resulting solid was dried under a high vacuum to afford a pure CuX-**1** complex, suitable for X-ray crystallography without further crystallization process. Further experimental details were provided in the Supplementary Information.

### Characterization

^1^H and ^13^C nuclear magnetic resonance (NMR) spectra were recorded on Bruker AVANCE NEO (500 MHz) and measured in CD_3_CN at the NMR facility of the Department of Chemistry, KAIST. Scanning electron microscopic (SEM) images were collected by using a field-emission SEM (FE-SEM) with FEI Insepct F50 at an acceleration voltage of 10.0 kV after Pt coating of samples by Cressington sputter coater 108auto for 45 s. Powder X-ray diffraction (PXRD) patterns were recorded on Rigaku SmartLab from 2*θ* = 4 to 60° with a scanning speed of 5° min^-1^ and step size of 0.01° from KAIST Analysis Center for Research Advancement (KARA). Fourier-transform infrared (FT-IR) spectra were recorded on Thermo Fischer Nicolet iS50 with attenuated total reflection (ATR) accessory from 400 to 4000 cm^−1^. Thermogravimetric (TGA) analysis was recorded on TA Instrument TGA Q50 at a heating rate of 10 °C ·min^−1^﻿ under the nitrogen atmosphere. Density functional theory (DFT) calculations were performed by Wavefunction Inc. Spartan 14^41^ software using Becke’s three-parameter Lee-Yang-Parr hybrid functional (B3LYP) with the 6-31** basis set. The model structures were identical to their crystal structures and were used without geometrical optimization. UV-Vis diffuse reflectance spectra were recorded on Perkin Elmer Lambda 1050 from 200 to 900 nm. The band gap energy was calculated using the Kubelka-Munk function^42,43^. Circular dichroism (CD) spectra were recorded on the JACSO J-815 spectrophotometer from KARA. Solid CD spectra were recorded from 190 to 360 nm with a scanning speed of 50 nm/min and were averaged 3 times. Photoluminescence (PL) spectra were recorded on Horiba LabRAM HR Evolution Visible NIR from 350 to 650 nm with a 325 nm excitation wavelength. PL lifetimes were measured on Horiba Fluorolog3 with time-correlated single-photon counting (TCSPC) with a 316 nm excitation wavelength and a ca. 550 nm emission range. The quantum yields of the obtained complexes were measured on Horiba Fluorolog3 equipped with an integrating sphere. Powder second-harmonic generation (SHG) spectra were recorded on modified Kurz-Perry nonlinear optical system following the literature procedure^35,44^. Ungraded samples of the reported compounds were packed into capillary tubes (1.8 mm internal diameter and 3.0 mm of outer diameter) and irradiated by a Q-switched Nd:YAG Beamtech Optronics Dawa-50 laser with a 1064 nm radiation. The SHG green light was collected by a Hamamatsu photomultiplier tube and monitored by a Tektronix TDS2012C oscilloscope. KH_2_PO_4_ (KDP) sample with a 150 to 200 μm particle size was used as a reference to compare the SHG efficiency. All numerical source data are available as Supplementary Data 5.

### Single crystal X-ray diffraction

Diffraction data were collected at the Pohang Accelerator Laboratory (PAL, Pohang-si, Gyeongsangbuk-do, Republic of Korea) synchrotron 2D-SMC beamline^45^ and 11C-Micro-MX beamline^46^ with monochromatic synchrotron radiation (λ = 0.700 Å). The collected images were indexed, integrated, and scaled using HKL-2000 and HKL-3000sm^47,48^. Space group determination and data reduction were performed by the XPREP program from the SHELXTL^49^ package. The structure was solved by direct methods with the SHELXT^50^ program and refined with the SHELXL^51^ embedded in Olex2^52,53^. For CuBr-**1**, TwinRotMat^54^ was used to handle non-merohedral twinning. For CuI-**1**, an empirical absorption correction by XABS2^55^ was performed to handle a deep electron density hole near the iodine atom. No restraints were used on the refinement.

### Supplementary information

Supplementary Information

Description of Additional Supplementary Files

Supplementary Data 1

Supplementary Data 2

Supplementary Data 3

Supplementary Data 4

Supplementary Data 5

## Data Availability

The authors declare that all additional data supporting the findings of this study are available within the Article itself and its Supplementary Information files and from the corresponding author upon request. For additional details regarding the experiment, please refer to the Supplementary Information. The X-ray crystallographic coordinates for structures reported in this Article have been deposited at the Cambridge Crystallographic Data Centre (CCDC) under deposition numbers 2041869 (CuCl-**1**, Supplementary Data 1), 2267125 (CuBr-**1**, Supplementary Data 2), 2041833 (CuBr-*ent*-**1**, Supplementary Data 3), and 2082900 (CuI-**1**, Supplementary Data 4). These data can be obtained free of charge from The Cambridge Crystallographic Data Centre via www.ccdc.cam.ac.uk/data_request/cif. CIF files are also available as Supplementary Data files 1–4. All numerical source data are available as Supplementary Data 5.
