# Supplementary material for: Cu(I)-thioether coordination complexes based on a chiral cyclic β-amino acid ligand
Source: Commun Chem. 2023 Nov 16;6:252. doi: 10.1038/s42004-023-01055-5 (PMC10654774; doi:10.1038/s42004-023-01055-5)
Supplement: Supplementary file 1 — Supplementary Information [file 42004_2023_1055_MOESM1_ESM.pdf]

Supplementary Information

Cu(I)-thioether coordination complexes based on a chiral cyclic β-amino acid ligand

Jihee Lee[1,2], Jaewook Kim[1,2], Hongil Jo[2,3] Danim Lim[1,2], Jungwoo Hong[1,2], Jintaek Gong[1,2,4], Kang Min Ok*[2,3], and Hee-Seung Lee*[1,2]

[1] Department of Chemistry, Korea Advanced Institute of Science and Technology (KAIST), 291 Daehak-ro, Yuseong-gu, Daejeon *34141*, Republic of Korea.

[2] Center for Multiscale Chiral Architectures (CMCA), KAIST, 291 Daehak-ro, Yuseong-gu, Daejeon *34141*, Republic of Korea.

[3] Department of Chemistry, Sogang University, 35 Baekbeom-ro, Mapo-gu, Seoul *04107*, Republic of Korea.

[4] Present Address: Department of Chemistry Education, Sunchon National University, 255 Jungang-ro, Suncheon-si, Jeollanam-do *57922*, Republic of Korea.

E-mail: hee-seung_lee@kaist.ac.kr and kmok@sogang.ac.kr

Supplementary Methods

Synthesis of CuCl-1 and CuCl-*ent*-1

150 mM of CuCl and 400 mM of **1**[1] in acetonitrile stock solutions were prepared. On the 4 mL vial, 1.0 mL of CuCl stock solution and 1.0 mL of **1** stock solution were mixed. After closing the cap tightly, the mixture was stirred at room temperature. A white precipitate formed immediately upon stirring. Solvents were decanted, and the resulting solid was dried under a high vacuum to afford 41 mg (31% based on CuCl) of pure CuCl-**1** complexes, suitable for X-ray crystallography without further crystallization process.

CuCl-*ent*-**1** was prepared from *ent*-**1** following the same procedure as CuCl-**1**. The crystalline solid was 28 mg (21% based on CuCl).

Synthesis of CuBr-1 and CuBr-*ent*-1

150 mM of CuBr and 400 mM of **1** in acetonitrile stock solutions were prepared. On the 4 mL vial, 1.0 mL of CuBr stock solution and 1.0 mL of **1** stock solution were mixed. After closing the cap tightly, the mixture was stirred at room temperature. A white precipitate formed immediately upon stirring. Solvents were decanted, and the resulting solid was dried under a high vacuum to afford 44 mg (32% based on CuBr) of pure CuBr-**1** complexes, suitable for X-ray crystallography without further crystallization process.

CuBr-*ent*-**1** was prepared from *ent*-**1** by following the same procedure as CuBr-**1**. 37 mg (27% based on CuCl), crystalline solid.

Synthesis of CuI-1 and CuI-*ent*-1

150 mM of CuI and 400 mM of **1** in acetonitrile stock solutions were prepared. On the 4 mL vial, 1.0 mL of CuI stock solution and 1.0 mL of **1** stock solution were mixed. After closing the cap tightly, the mixture was stirred at room temperature. A white precipitate formed immediately upon stirring. Solvents were decanted, and the resulting solid was dried under a high vacuum to afford 29 mg (42% based on CuI) of pure CuI-**1** complexes, suitable for X-ray crystallography without further crystallization process.

CuI-*ent*-**1** was prepared from *ent*-**1** by following the same procedure as CuI-**1**. 31 mg (46% based on CuCl), crystalline solid.

3. Supplementary Figures

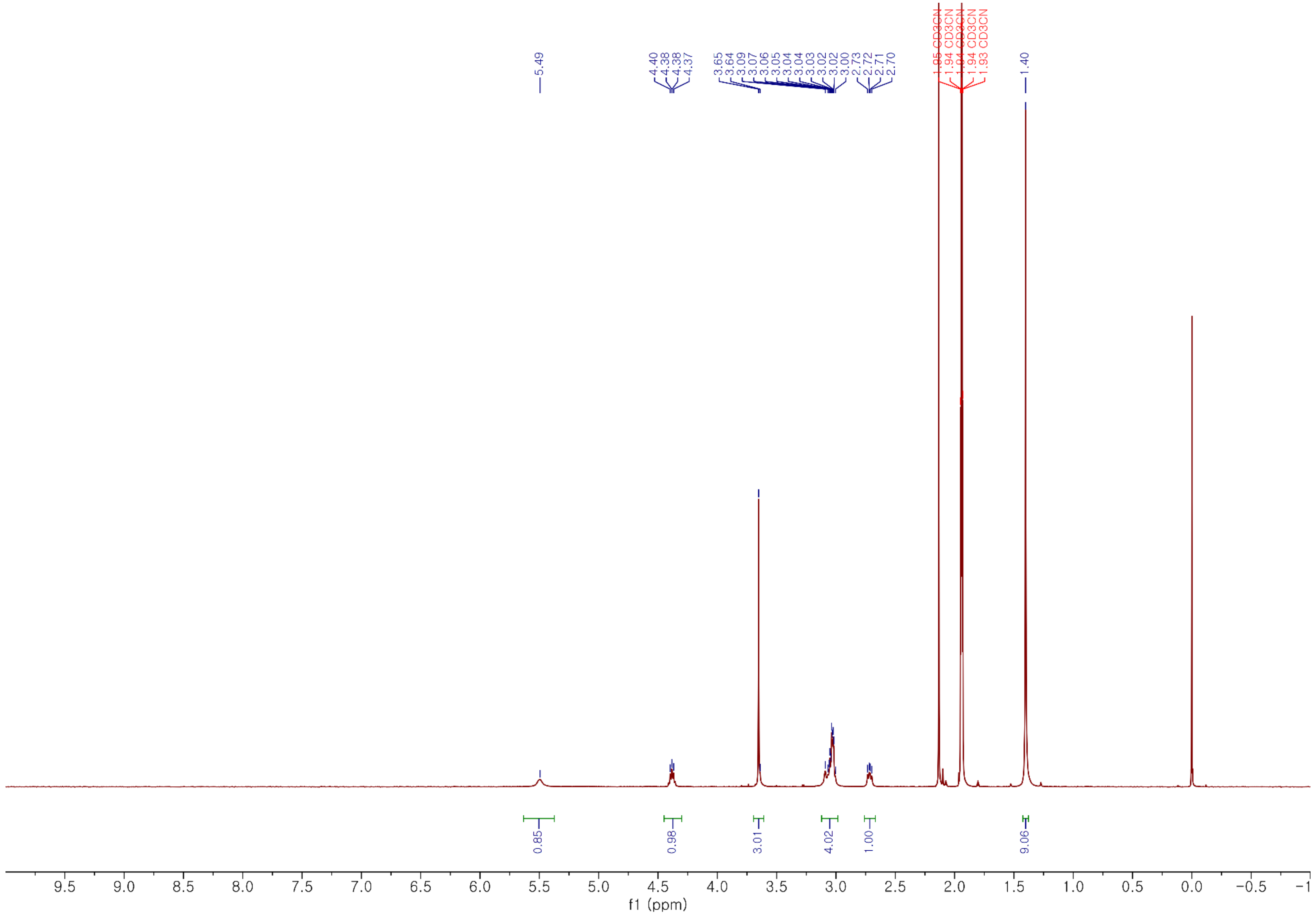

Supplementary Figure 1. ^{1}H NMR spectrum (500 MHz, 298 K, CD_3CN) of CuCl-**1**.

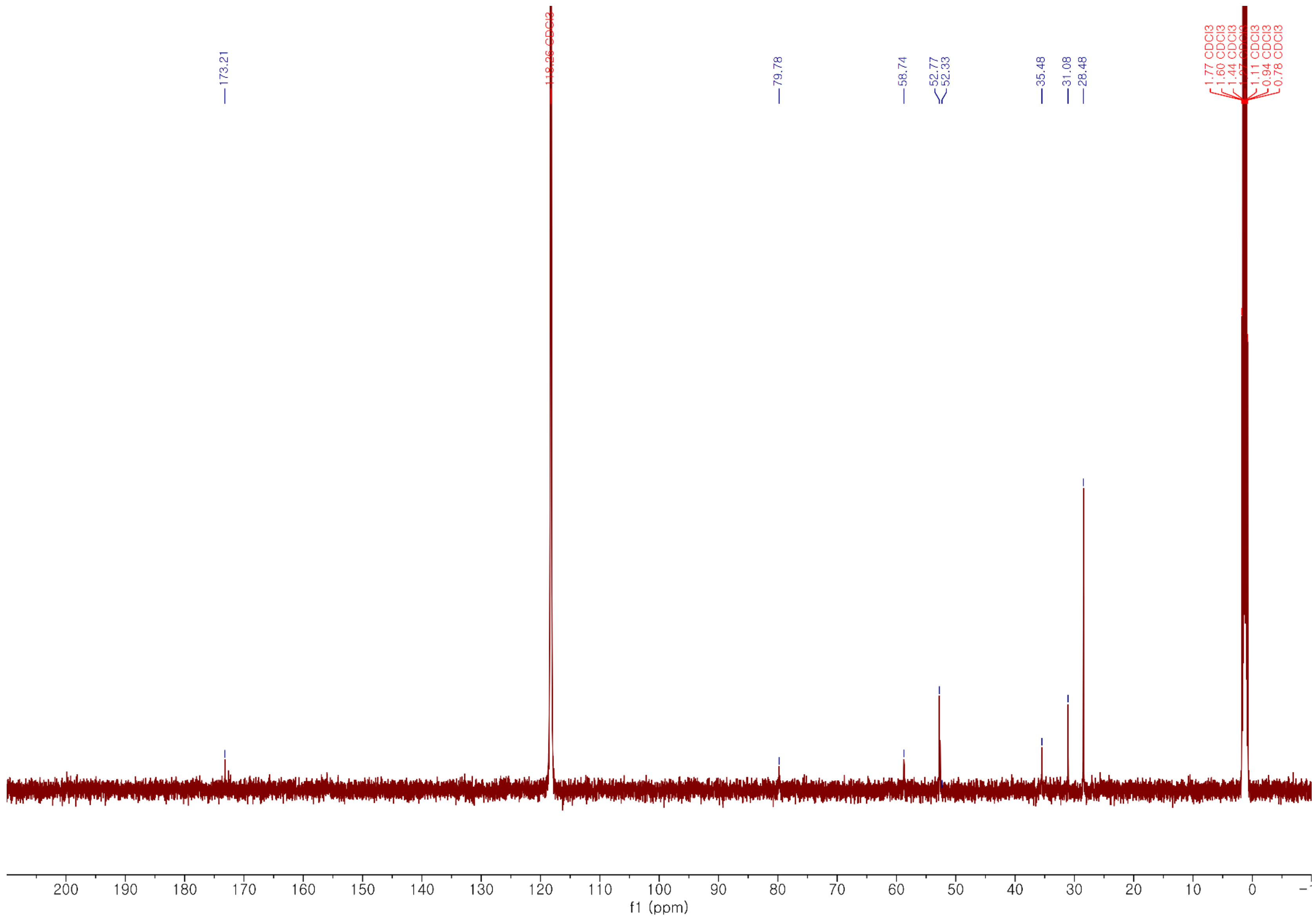

Supplementary Figure 2. ^{13}C NMR spectrum (126 MHz, 298 K, CD_3CN) of CuCl-**1**.

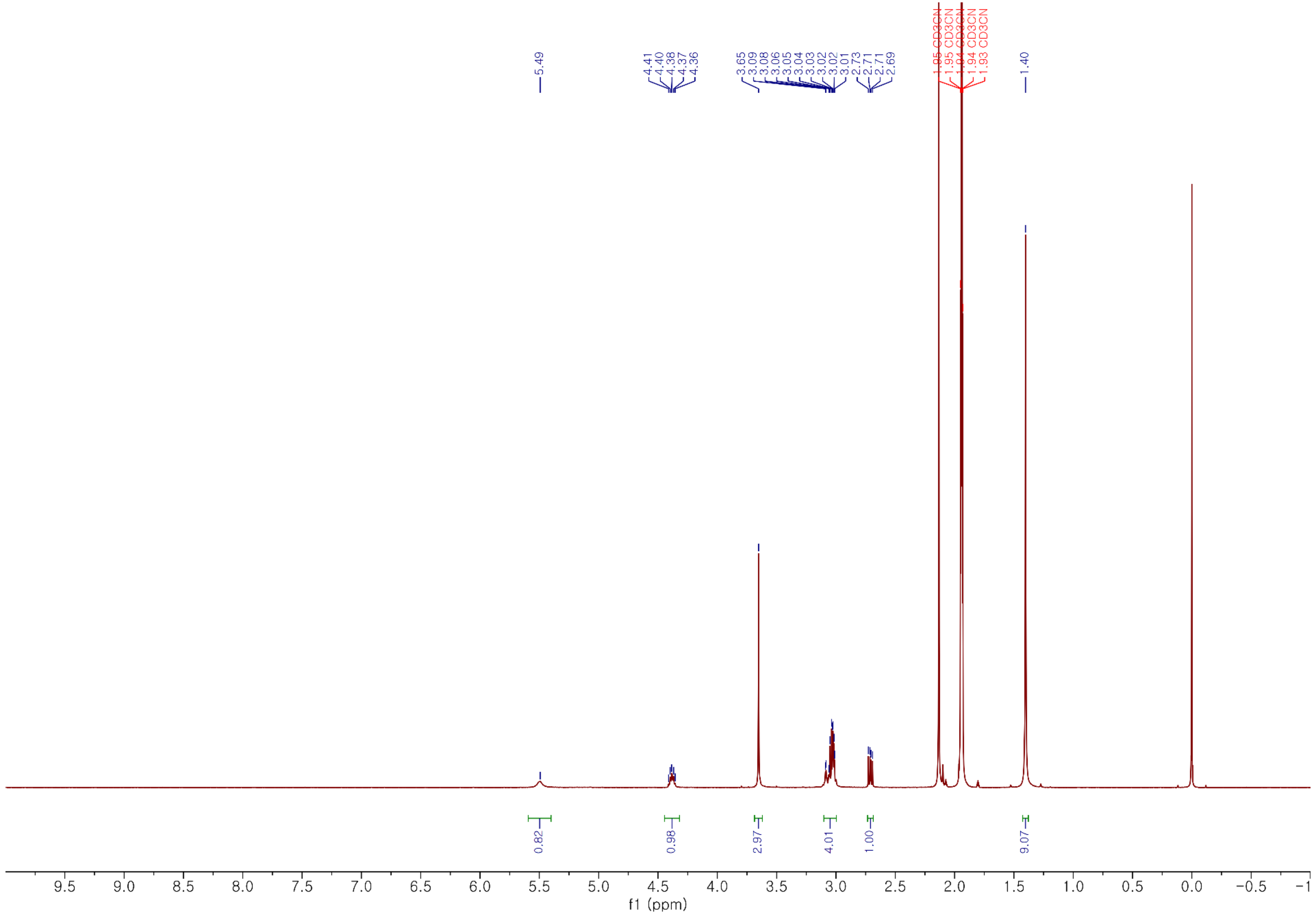

Supplementary Figure 3. ^{1}H NMR spectrum (500 MHz, 298 K, CD_3CN) of CuBr-**1**.

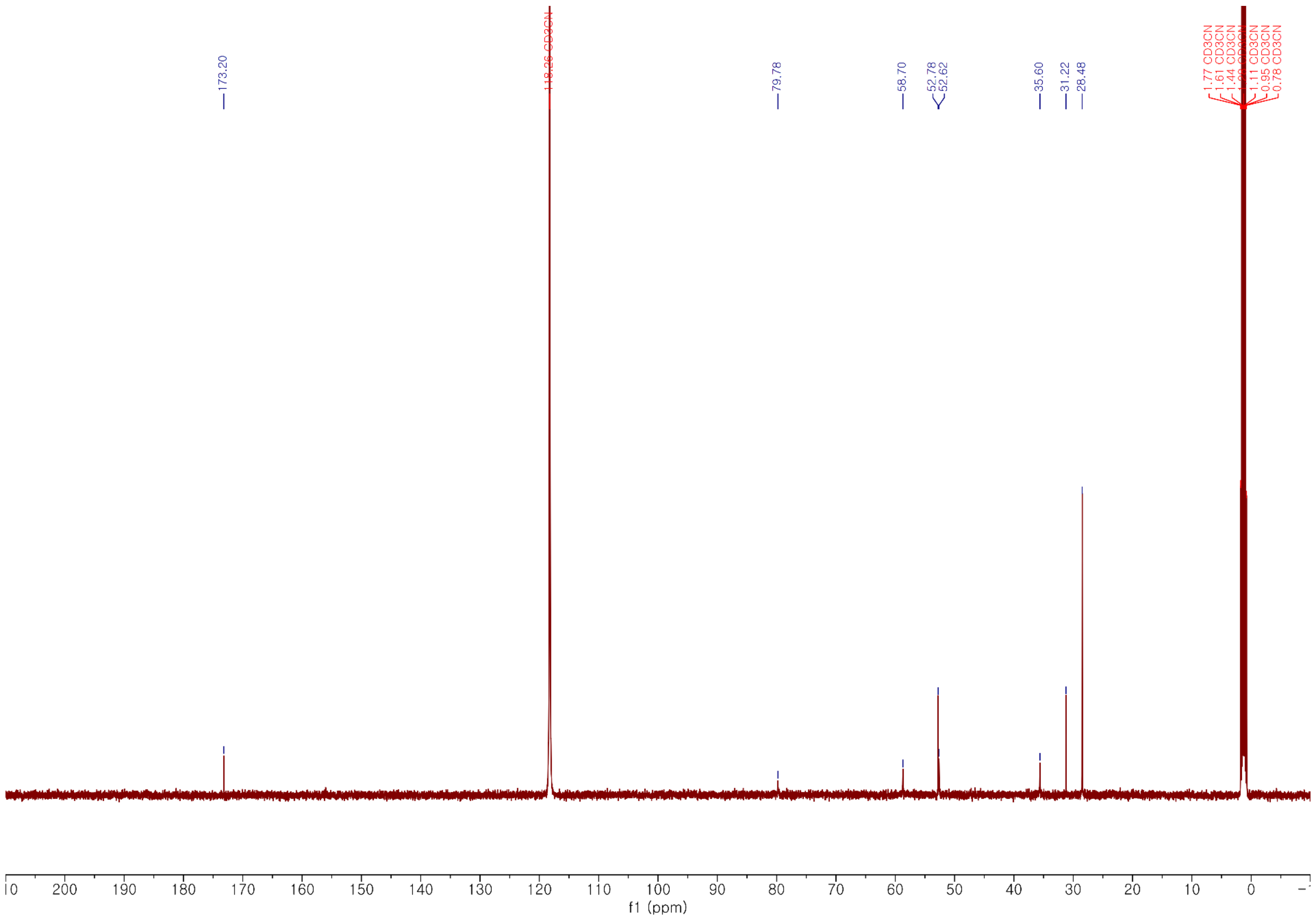

Supplementary Figure 4. ^{13}C NMR spectrum (126 MHz, 298 K, CD_3CN) of CuBr-**1**.

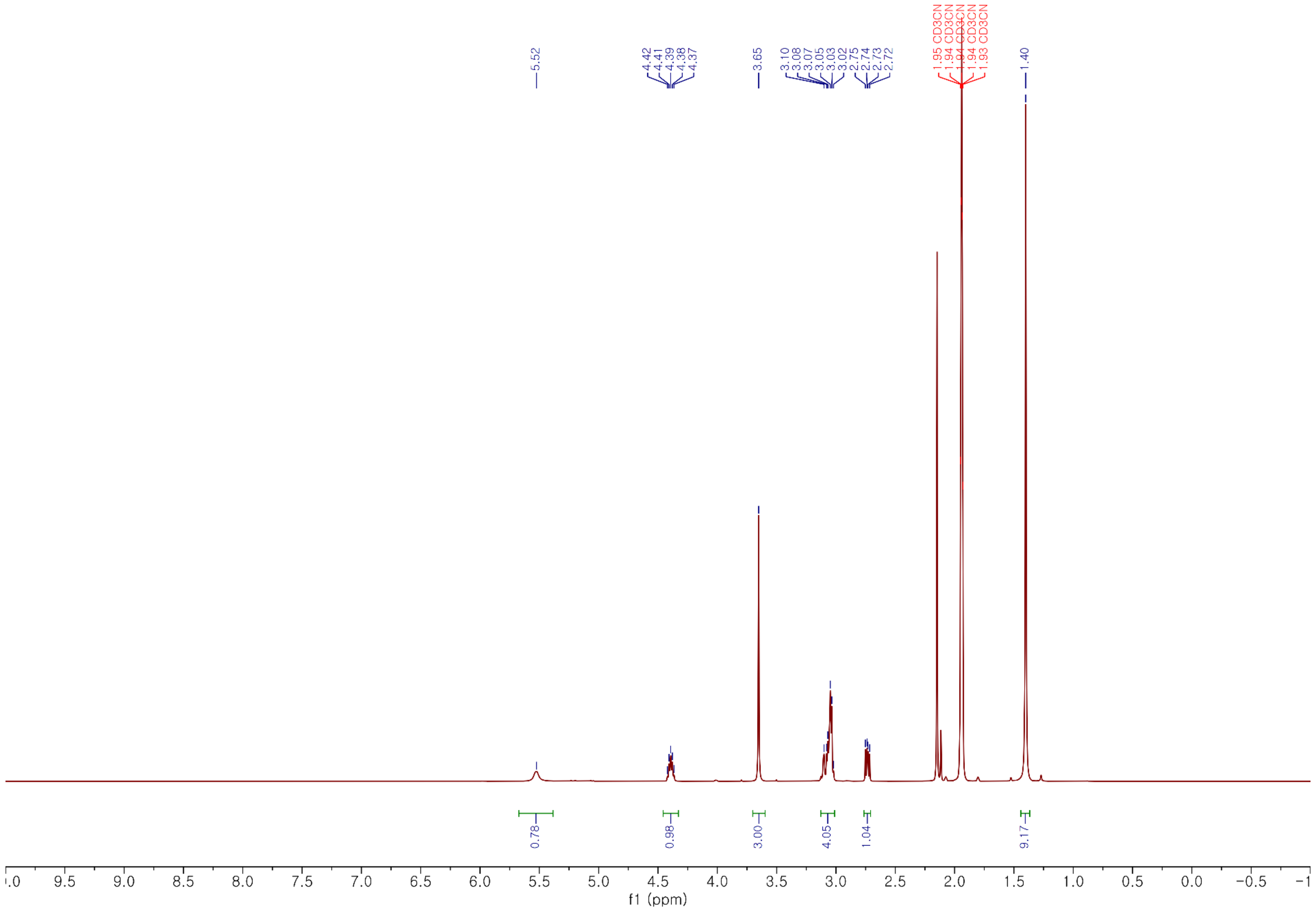

Supplementary Figure 5. ^{1}H NMR spectrum (500 MHz, 298 K, CD_3CN) of CuI-**1**.

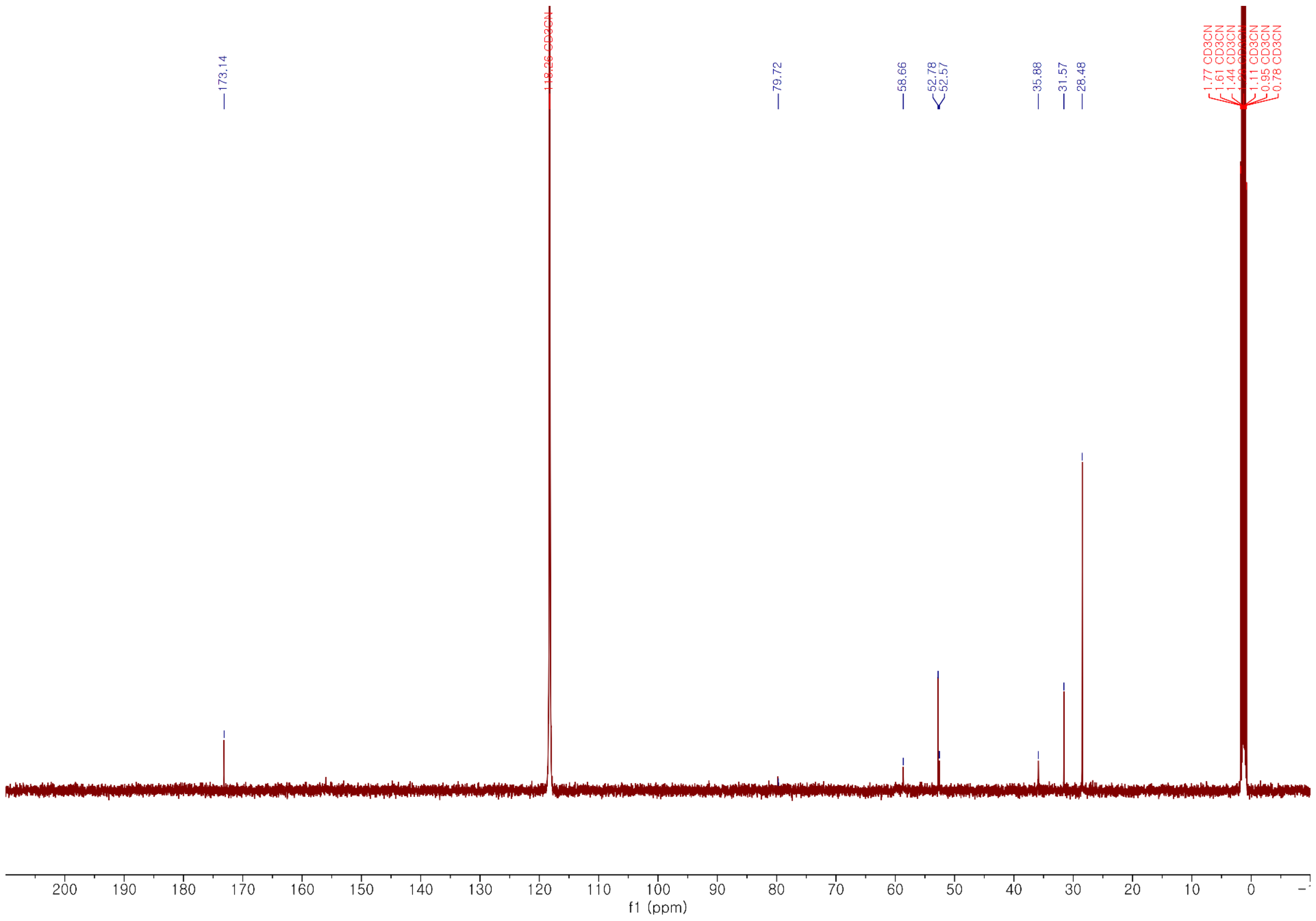

Supplementary Figure 6. ^{13}C NMR spectrum (126 MHz, 298 K, CD_3CN) of CuI-**1**.

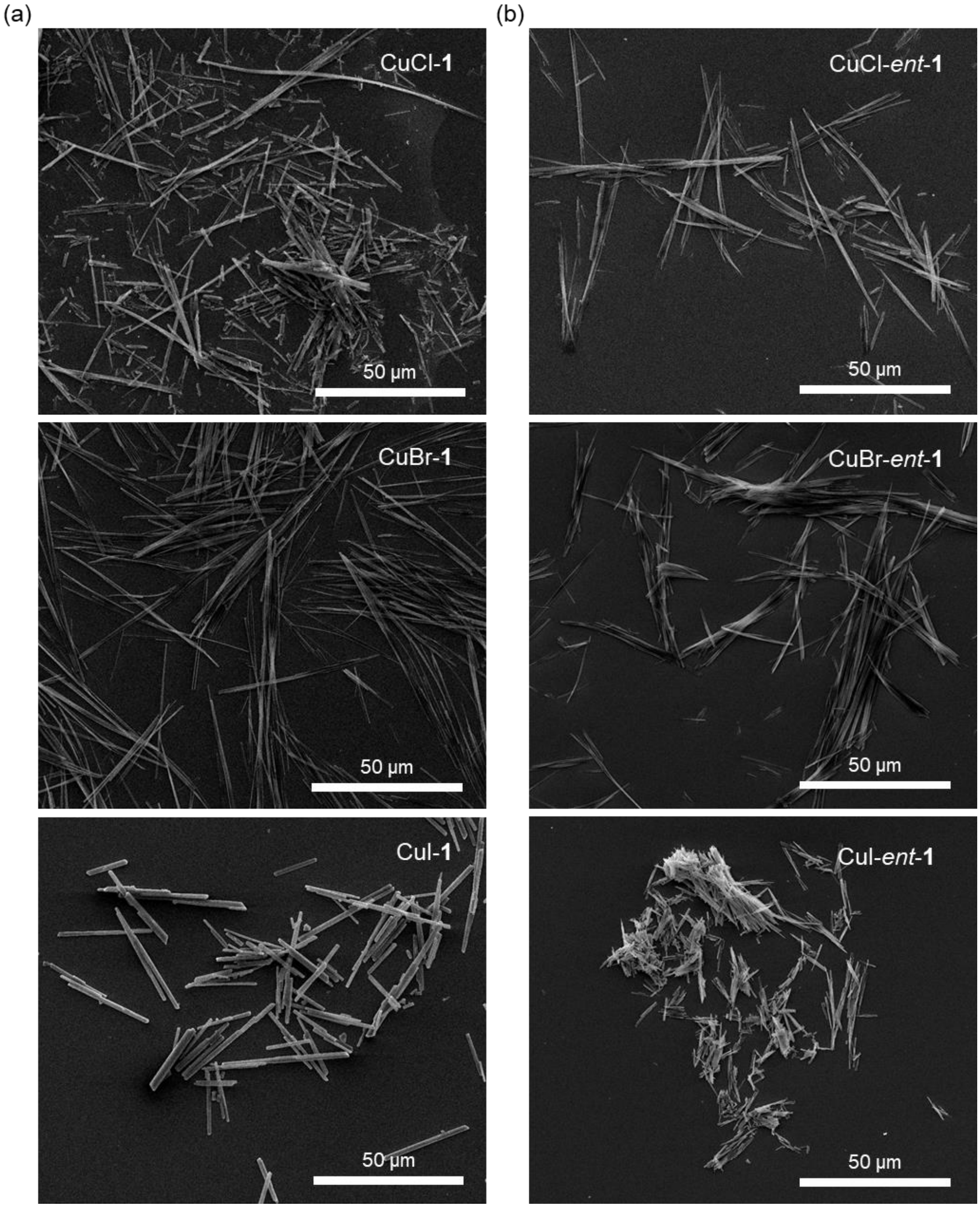

Supplementary Figure 7. Scanning electron microscope (SEM) images of (a) CuX-**1** and (b) CuX-*ent*-**1** (X = Cl, Br, and I) complexes.

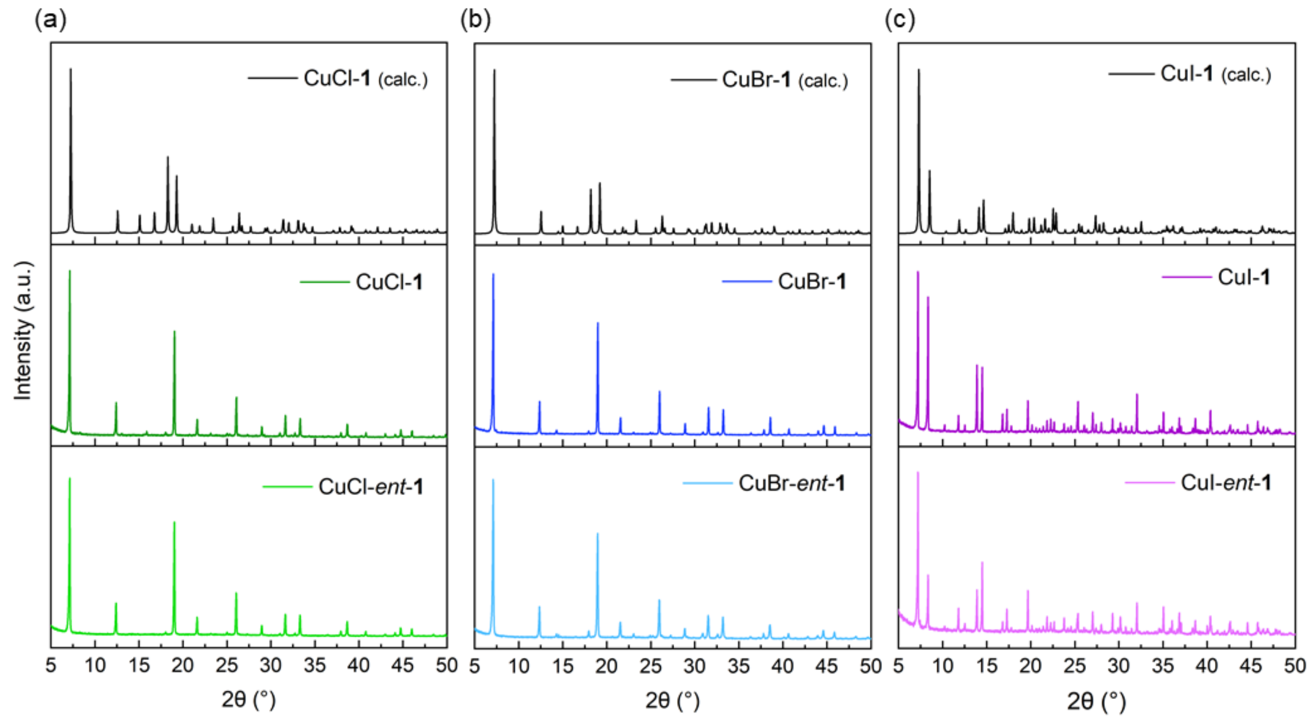

Supplementary Figure 8. Calculated and experimental powder X-ray diffraction (PXRD) patterns of (a) CuCl-ATTC, (b) CuBr-ATTC, and (c) CuI-ATTC.

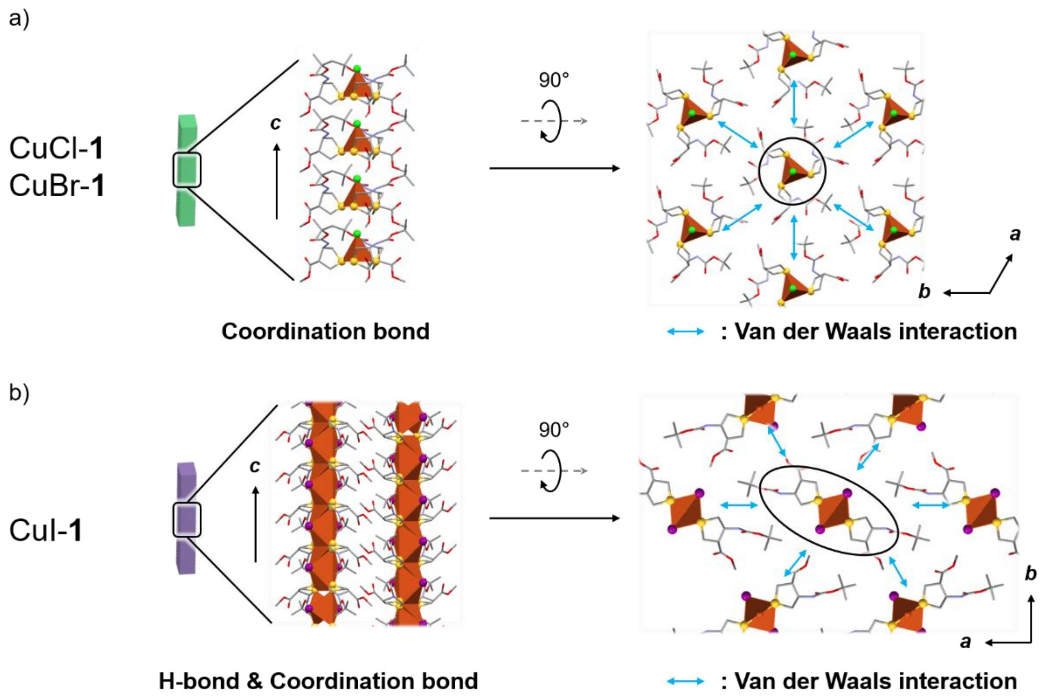

Supplementary Figure 9. Molecular arrangement in needle-shaped crystals (a) CuCl-**1**, CuBr-**1**, and (b) CuI-**1** with an indication of involved non-covalent bond interactions.

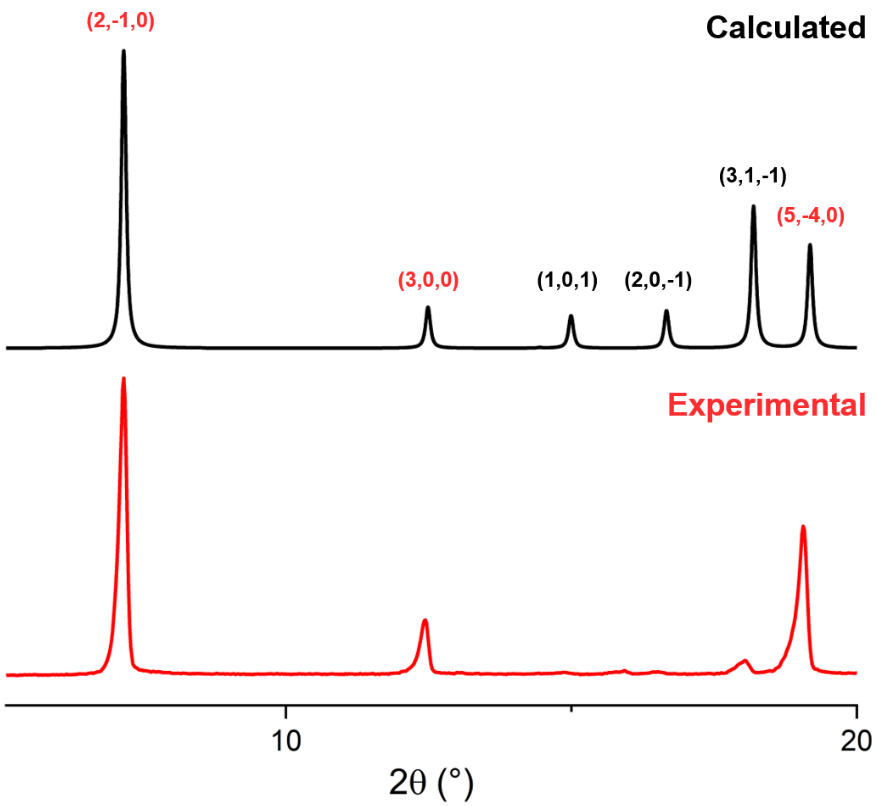

Supplementary Figure 10. Detailed comparison between (top) calculated and (down) experimental PXRD pattern of CuX-**1** (X = Cl, Br). Since CuCl-**1** and CuBr-**1** share the same crystal structure, the PXRD pattern of CuCl-**1** was used for comparison. Some Miller indices (displayed in black) were observed only in the calculated PXRD pattern.

Supplementary Discussion: Difference between Calculated and Experimental PXRD Patterns

The SEM images of all CuX-**1** (X = Cl, Br, I) revealed needle-shaped crystals, indicating a strong 1D molecular interaction inducing single crystal growth along a typical direction. The packing structure of CuCl-**1** and CuBr-**1** shows dipole-dipole interaction between the Cu-X bond through the *c*-axis (Figure S3a). On the other hand, the packing structure of the CuI-**1** also shows hydrogen bonds and coordination bonds along the *c*-axis (Figure S3b). Meanwhile, the other axis of CuX-**1** involves Van der Waals interaction, which is weaker and less directional. Moreover, the PXRD pattern for CuCl-**1** and CuBr-**1** showed significantly declined intensities of Miller indices involving the *c*-axis (Figure S4). This strong preferred orientation indicates that the c-axis is aligned with the long axis of the needle-shaped crystals. After all, this evidence supports that CuX-**1** (X = Cl, Br, I) complexes prefer crystal growth toward the *c*-axis.[18,19]

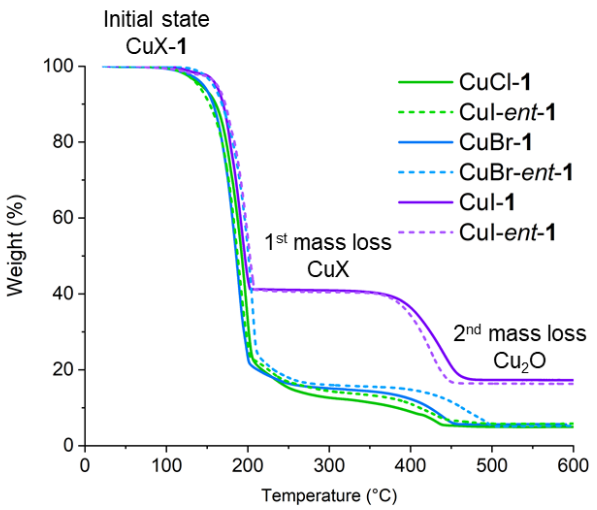

Compound	1st mass loss (300 ℃)	2nd mass loss (500 ℃)
CuCl-**1** (theoretical)	88.8%	91.9%
CuCl-**1**	87.3%	95.0%
CuCl-*ent*-**1**	85.6%	94.2%
CuBr-**1** (theoretical)	84.5%	92.3%
CuBr-**1**	84.9%	94.4%
CuBr-*ent*-**1**	84.0%	94.8%
CuI-**1** (theoretical)	57.8%	84.2%
CuI-**1**	60.0%	82.6%
CuI-*ent*-**1**	59.5%	83.6%

Supplementary Figure 11. (left) Thermogravimetric (TG) analysis spectra of the CuX-**1** and CuX-*ent*-**1** (X = Cl, Br, I) complexes spanning from room temperature to 600 ℃ under flowing air. (right) Theoretical and experimental mass loss of CuX-**1** and CuX-*ent*-**1** (X = Cl, Br, I) complexes.

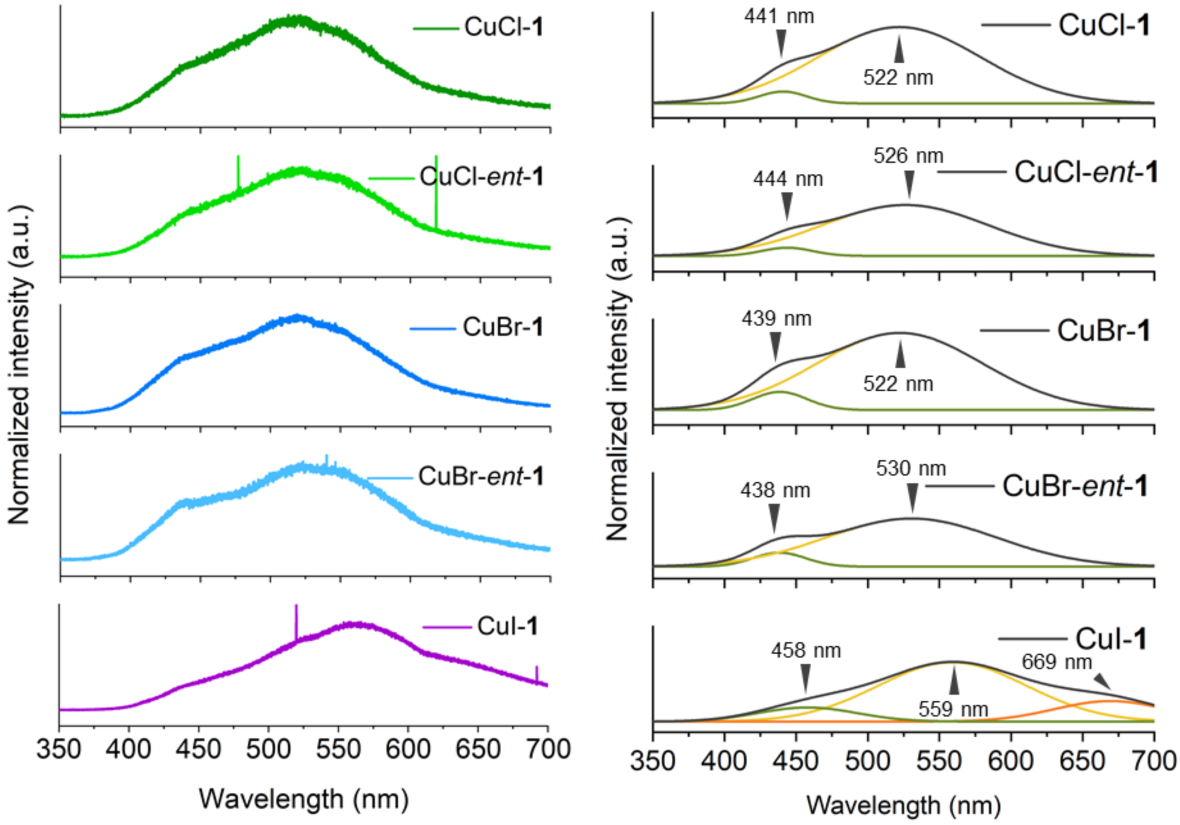

Supplementary Figure 12. (left) Solid-phase photoluminescence (PL) spectra of the CuX-**1** and CuX-*ent*-**1** (X = Cl, Br, I) complexes at 293 K. (right) Smoothed and deconvoluted PL spectra.

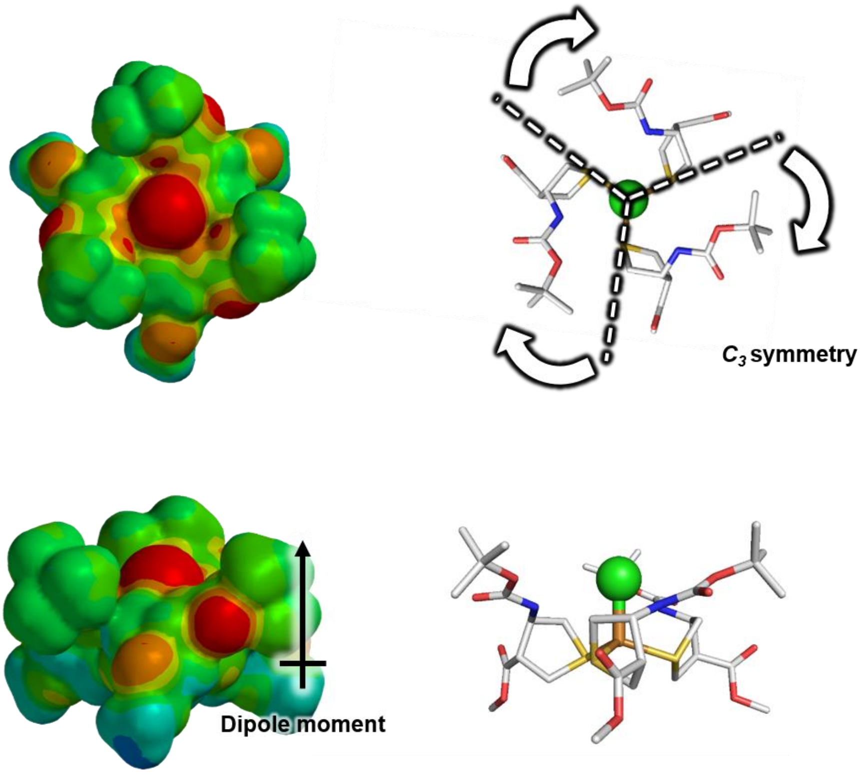

Supplementary Figure 13. Dipole moment of CuX-**1** complex (X = Cl, Br). (left) Electrostatic potential map of CuX-ATTC. The electrostatic potential value is color-scaled with negative in red and positive in blue. (right) Crystal structure of CuX-ATTC with the same view of the electrostatic potential map. The halide atom is indicated in a green ball, while other atoms are indicated in the stick model.

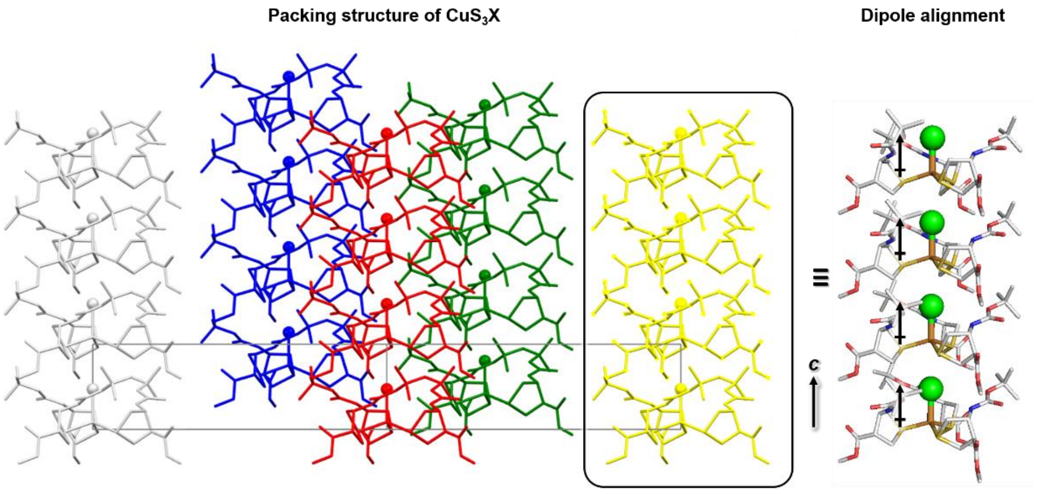

Supplementary Figure 14. Dipole alignment of CuX-ATTC (X = Cl, Br) complex.

Supplementary Table 1. Crystallographic Information

Compound	CuCl-**1**	CuBr-**1**	CuBr-*ent*-**1**	CuI-**1**
CCDC Deposition #	2041869	2267125	2041833	2082900
Empirical formula	$C_{11}H_{19}Cl_{0.33}Cu_{0.33}NO_4S$	$C_{11}H_{19}Br_{0.33}Cu_{0.33}NO_4S$	$C_{11}H_{19}Br_{0.33}Cu_{0.33}NO_4S$	$C_{11}H_{19}CuINO_4S$
Formula weight (g/mol)	294.33	309.15	309.15	451.77
Temperature (K)	100.0	100.0	100.0	100.0
Crystal system	trigonal	trigonal	trigonal	orthorhombic
Space group	*R*3	*R*3	*R*3	$P2_12_12$
a (Å)	24.339(3)	24.422(3)	24.427(3)	14.885(3)
b (Å)	24.339(3)	24.422(3)	24.427(3)	20.713(4)
c (Å)	6.1060(12)	6.1470(12)	6.1480(12)	5.0730(10)
α (°)	90	90	90	90
β (°)	90	90	90	90
γ (°)	120	120	120	90
Volume (Å^3)	3132.4(11)	3175.1(11)	3177.0(11)	1564.1(5)
Z	9	9	9	4
ρ_{calc} (g/cm^3)	1.404	1.455	1.454	1.919
μ (mm^{-1})	0.766	1.599	1.598	3.380
F(000)	1398.0	1452.0	1452.0	888.0
Crystal size (mm^3)	0.12 × 0.01 × 0.01	0.2 × 0.016 × 0.016	0.37 × 0.006 × 0.006	0.128 × 0.017 × 0.009
Radiation	synchrotron (λ = 0.700)	synchrotron (λ = 0.700)	synchrotron (λ = 0.700)	synchrotron (λ = 0.700)
2θ range for data collection (°)	3.296 to 66.624	3.284 to 66.598	3.284 to 52.962	3.318 to 60.054
Index ranges	$-34 \le h \le 34$, $-34 \le k \le 35$, $-8 \le l \le 8$	$-37 \le h \le 35$, $-36 \le k \le 37$, $-8 \le l \le 8$	$-31 \le h \le 31$, $-31 \le k \le 31$, $-7 \le l \le 7$	$-21 \le h \le 21$, $-29 \le k \le 29$, $-7 \le l \le 7$
Reflections collected	11383	4876	7760	15221
Independent reflections	4330 [R_{int} = 0.1484, R_{sigma} = 0.1596]	4876 [R_{int} = , R_{sigma} = 0.1751]	2977 [R_{int} = 0.1169, R_{sigma} = 0.1002]	4773 [R_{int} = 0.0665, R_{sigma} = 0.0594]
Data/restraints/parameters	4330/1/164	4876/1/165	2977/13/159	4773/0/195
Goodness-of-fit on F^2	0.904	1.034	1.139	0.949
Final *R* indexes [I>=2σ (I)]	R_1 = 0.0556, wR_2 = 0.0971	R_1 = 0.0667, wR_2 = 0.1418	R_1 = 0.0521, wR_2 = 0.1286	R_1 = 0.0332, wR_2 = 0.0724
Final *R* indexes [all data]	R_1 = 0.1249, wR_2 = 0.1196	R_1 = 0.1313, wR_2 = 0.1800	R_1 = 0.0712, wR_2 = 0.1535	R_1 = 0.0475, wR_2 = 0.0761
Largest diff. peak/hole / e (Å^{-3})	0.67/-1.04	1.46/-1.54	0.93/-1.19	0.69/-0.65
Flack parameter	-0.016(17)	0.040(12)	0.009(8)	-0.011(13)

Supplementary Table 2. Selected bond lengths and bond angles observed in crystal structures.

Crystal structure		Bond	Bond length (Å)	Bond	Bond angle (°)
CuCl-**1**		Cu–S	2.312	S–Cu–S	110.73
		Cu–Cl	2.352	S–Cu–Cl	108.18
		N–Cl	3.391	N–Cl–Cu	71.86
CuBr-**1**		Cu–S	2.319	S–Cu–S	111.20
		Cu–Br	2.482	S–Cu–Br	107.68
		N–Br	3.501	N–Br–Cu	70.14
CuBr-*ent*-**1**		Cu–S	2.317	S–Cu–S	111.07
		Cu–Br	2.473	S–Cu–Br	107.82
		N–Br	3.501	N–Br–Cu	70.00
CuI-**1**	rhomboid A	Cu–S	2.386, 2.361	S–Cu–S	106.05, 102.70
		Cu–I	2.709, 2.647	S–Cu–Cu	126.98, 128.65
		Cu–Cu	2.823	I–Cu–I	114.27, 118.52
	rhomboid B	Cu–S	2.304, 2.277	S–Cu–S	148.90
		Cu–I	2.582, 2.625	S–Cu–Cu	104.50, 106.28
		Cu–Cu	2.531	I–Cu–I	121.84

Supplementary Table 3. Luminescence parameter for CuX-ATTC complexes (X = Cl, Br, I).

Compound	T (K)	λ_{em} (nm)	Stokes shift (cm^{-1})	Lifetime (ns)	Quantum yield (%)
CuCl-**1**	293	522	11500	19.45	2.44
CuCl-*ent*-**1**	293	526	11600	19.89	1.36
CuBr-**1**	293	522	11500	19.68	2.12
CuBr-*ent*-**1**	293	530	11600	19.33	3.79
CuI-**1**	293	559	13000	35.71	1.20

Supplementary References

1. Lim, D. *et al*. Versatile post-synthetic modifications of helical β-peptide foldamers derived from a thioether-containing cyclic β-amino acid. *Angew. Chem. Int. Ed.*, **62**, e202305196 (2023).

2. Dollase, W. A. Correction of intensities for preferred orientation in powder diffractometry: application of the March model. *J. Appl. Cryst.* **19**, 267–272 (1986).

3. Gong, J., Eom, J.-H., Jeong, R., Driver, R. W. & Lee H.-S. Structural analysis of the foldecture derived from racemic peptide foldamers. *Solid State Sci.* **70**, 1–5 (2017).
