# Supplementary material for: Cu(I)-thioether coordination complexes based on a chiral cyclic β-amino acid ligand
Source: Commun Chem. 2023 Nov 16;6:252. doi: 10.1038/s42004-023-01055-5 (PMC10654774; doi:10.1038/s42004-023-01055-5)
Supplement: Supplementary file 2 — Description of Additional Supplementary Files [file 42004_2023_1055_MOESM2_ESM.pdf]

Description of Additional Supplementary Files

File name: Supplementary Data 1

Description: CIF file for CuCl-1

File name: Supplementary Data 2

Description: CIF file for CuBr-1

File name: Supplementary Data 3

Description: CIF file for CuBr-ent-1

File name: Supplementary Data 4

Description: CIF file for CuI-1

File name: Supplementary Data 5

Description: : Numerical source data
